# Design of Monovalent Ion Selective Membranes for Reducing the Impacts of Multivalent Ions in Reverse Electrodialysis

**DOI:** 10.3390/membranes10010007

**Published:** 2019-12-31

**Authors:** Abreham Tesfaye Besha, Misgina Tilahun Tsehaye, David Aili, Wenjuan Zhang, Ramato Ashu Tufa

**Affiliations:** 1 Department of Chemistry, College of Natural and Computational Science, Jigjiga University, P.O. Box 1020, Jigjiga, Ethiopia; abrehamtesfaye396@gmail.com; 2 Univ. Grenoble Alpes, Univ. Savoie Mont Blanc, CNRS, Grenoble INP, LEPMI, 38 000 Grenoble, France; misginabcen@gmail.com; 3 Department of Energy Conversion and Storage, Technical University of Denmark, Building 310, 2800 Kgs. Lyngby, Denmark; larda@dtu.dk; 4 School of Environmental and Municipal Engineering, Tianjin Chengjian University, Tianjin 300384, China; wenjuanvivian@126.com

**Keywords:** salinity gradient power, reverse electrodialysis, uphill transport, monovalent selective membranes, multivalent ions

## Abstract

Reverse electrodialysis (RED) represents one of the most promising membrane-based technologies for clean and renewable energy production from mixing water solutions. However, the presence of multivalent ions in natural water drastically reduces system performance, in particular, the open-circuit voltage (OCV) and the output power. This effect is largely described by the “uphill transport” phenomenon, in which multivalent ions are transported against the concentration gradient. In this work, recent advances in the investigation of the impact of multivalent ions on power generation by RED are systematically reviewed along with possible strategies to overcome this challenge. In particular, the use of monovalent ion-selective membranes represents a promising alternative to reduce the negative impact of multivalent ions given the availability of low-cost materials and an easy route of membrane synthesis. A thorough assessment of the materials and methodologies used to prepare monovalent selective ion exchange membranes (both cation and anion exchange membranes) for applications in (reverse) electrodialysis is performed. Moreover, transport mechanisms under conditions of extreme salinity gradient are analyzed and compared for a better understanding of the design criteria. The ultimate goal of the present work is to propose a prospective research direction on the development of new membrane materials for effective implementation of RED under natural feed conditions.

## 1. Introduction

The ever-increasing demand for energy due to population growth, industrialization, and urban area expansion along with the fossil fuel runout drives the need for a vigorous energy supply toward sustainable growth and improved living standards. Primary energy consumption is growing at a fast rate, reported to be 2.9% in 2018, which was almost double the 10-year average of 1.5% per year [1]. This energy consumption was mostly driven by natural gas, which is associated with considerable greenhouse gas emissions. In this regard, the development of alternative energy resources that satisfy the demand for clean energy and related environmental issues is urgently required. Salinity gradient energy, also referred to as “blue energy” is the energy obtained by the conversion of the chemical potential difference to electrical/mechanical energy by mixing two different salt solutions [2,3]. It is an entirely clean energy source with no toxic gas emissions and no environmental impact. The flow of 1 m^3^ of freshwater into the sea produces around 0.8 kWh theoretical energy. In this sense, the total freshwater flow of the major rivers into the sea generates nearly 2 TW of salinity gradient power (SGP) [3,4]. Of the total global potential of SGP (2 TW), about 50% (0.98 TW) is estimated to be available for extraction. While seawater and rivers remain the most commonly used feed solutions for harnessing SGP, other feed sources, such as brine solutions from anthropogenic activities (e.g., solar ponds or membrane desalination), natural sources (e.g., salt lakes), and thermolytic solutions, could also be employed to produce SGP. 

**Figure 1:**
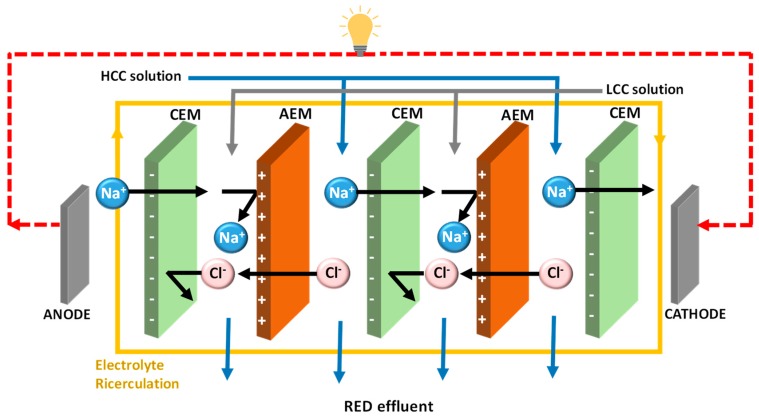
Schematic illustration of reverse electrodialysis (RED) for salinity gradient power generation. The high concentration compartment (HCC) and low concentration compartment (LCC) are created by a series of alternative cation exchange membranes (CEMs) and anion exchange membranes (AEMs). The electrical energy is generated by the redox reactions occurring over the two electrodes placed at the ends of the membrane pile.

One of the most promising membrane-based technologies for harnessing SGP is reverse electrodialysis (RED), which is currently at an advanced stage of development. In RED, cation exchange membranes (CEMs) and anion exchange membranes (AEMs) are aligned in alternative ways to create a series of adjacent compartments, termed as “high concentration compartments (HCCs)” and “low concentration compartments (LCCs)” (Figure 1). The compartments are separated by spacer materials which provide a space between the membranes, thereby allowing mixing of the salt solutions. When these compartments are supplied with respective high concentration solutions and low concentration solutions, a potential gradient is created that drives the selective transport of ions through the membranes. The electrode connected to the external circuit allows conversion of the ionic flux into electricity [3].

The effectiveness of RED and its technological progress in real practice (i.e., using natural feeds) is hampered by the unavailability of a suitable selective ion exchange membrane (IEM). Additionally, a large part of RED investigations is limited to artificial aqueous solutions containing only NaCl. However, natural water solutions contain other ions, including multivalent ions which exhibit a negative impact on RED performance [5,6]. Natural seawater contains more than 10% by weight of multivalent ions such as Mg^2+^, Ca^2+^, and SO_4_^2−^ [5,7]. Several research works were carried out to study the effect of multivalent ions on RED equipped with different commercial membranes [5,6,7,8,9]. Overall, the presence of multivalent ions resulted in a reduction of the open-circuit voltage (OCV) and the power density (*P_d_*). The decreases in the *P_d_* and the OCV were centrally associated with the increase in the IEM resistance and decreased the permselectivity [3]. Furthermore, the occurrence of the multivalent ions transported against the concentration gradient created an uphill transport [7]. 

Several review papers presented important achievements in RED, covering topics on membrane development [3,10,11], stack design and fluid dynamics [3], process optimization and impact of operation conditions [3,4], electrochemical and physical properties [11], membrane fouling, etc. [3]. Specifically, Luo et al. reviewed the structure of an IEM and ion transport and methods to improve IEM selectivity in (electrodialysis) ED [12]. Ge et al. provided an update on the advances of the monovalent cation perm-selective membrane for ED [13]. Recently, Tufa et al. performed a more comprehensive review of RED [3]. Veerman et al. reviewed the fundamentals of RED, including the process analysis, the stack benchmarking methods, and research development [14]. However, there are still gaps concerning monovalent ion-selective membrane applications for RED, with a particular focus on limiting the impact of multivalent ions. In the present work, we critically review the impacts of multivalent ions on power generation by RED along with the effect of the feed salinity conditions. We systematically analyze the use of a monovalent selective IEM as an alternative potential solution to reduce the negative impact of multivalent ions for SGP in RED application. Moreover, the challenges associated with IEMs, such as the availability of low-cost materials and its synthesis methodologies, are discussed. Finally, we provide a prospective on the application of conducting polymers for RED.

## 2. Transport Phenomenon in RED

The transport phenomenon in RED is principally governed by the transport of the ions in the membranes and solutions. This consists of the electro-migrative flux of ions to and toward the surface and within IEMs and the convective flux through spacer channels along IEMs [3,15]. In an ideal situation, only counter-ions would pass through the membranes, with the co-ions and the water being rejected. However, co-ions and water can cross the membrane, thereby reducing the performance of RED [3,15,16]. Though modeling of the ion transport phenomenon in RED/ED is a complex task, several authors described ion transport using different modeling approaches, such as Nernst–Planck transport [15,16], irreversible thermodynamics formalism [17], the Stefan–Maxwell theory [18], and the semi-empirical model [19]. The transport phenomenon in RED becomes even more complex when a mixture of solutions is used instead of the traditional NaCl salt solution [5]. Before looking into the details of ion transport, it is necessary to understand some of the fundamental performance parameters of RED.

### 2.1. Key Performance Parameters of RED

The total voltage in RED under the open circuit conditions is termed open-circuit voltage (OCV), which is the sum of the total Nernst potential drop over each membrane. Theoretically, OCV is calculated by the Nernst equation, shown by Equation (1).

(1) $$\mathrm{OCV}\;=\;\frac{NRT}{F}\;\left[\frac{\alpha_{CEM}}{z_{cn}}ln\frac{\gamma_{c}C_{c}}{\gamma_{d}C_{c}}+\frac{\alpha_{AEM}}{Z_{an}}ln\frac{\gamma_{c}C_{c}}{\gamma_{d}C_{d}}\right]$$

where *N* is the number of membrane pairs (cell) pairs, *γ* is the activity coefficient of the ions, *R* is the universal gas constant (8.314 J/K mole), *F* is the Faraday’s constant (96,500 C/mole), *T* is the temperature (K), *α* is the permselectivity of the ion exchange membrane, *Z* is the ionic valence, the subscripts “*cn*” and “*an*” stand for “anion” and “cation”, whereas the subscripts “*c*” and “*d*” stand for “concentrate” and “dilute”, respectively. Membrane permselectivity highly influences the OCV. The permselectivity is basically defined as the ability of the membrane to selectively transport only counter-ions (e.g., anions for AEM) and exclude co-ions (e.g., cations for AEM) [3,4]. The higher the permselectivity*,* the higher the OCV (see Equation (1)). Furthermore, the concentration gradient and the valence of the transported-ions also affect the OCV. The value of permselectivity is calculated as the ratio of the measured electrical potential (*E*_m_) under a given concentration gradient and the theoretical potential (*E_t_*):

(2) $$\alpha_{app\;}=\;\frac{E_{m}}{E_{t}}$$

In RED, the internal stack resistance *R*_i_ (Ω) is the sum of the ohmic resistance (*R*_ohmic_), the non-ohmic resistance (*R*_non-omic_), and the resistance of the electrode system (*R*_el_). The R_non-omic_ is the sum of the resistance from the electrical double layer (*R*_edl_) and the diffusion boundary layer (*R*_dbl_); it is usually very low for concentrated feed solution [2,3,4,11].

*R*_i_ (Ω) = *R*_ohmic_ + *R*_non-omic_ + *R*_el_ (3)

The *R*_ohmic_ of an RED is the sum of the resistance of the membrane (*R*_IEM_) and the resistance of feed solutions. The *R*_IEM_ is the sum of the *R*_AEM_ and the *R*_CEM_. Thus, the total resistance in RED can also be represented as

*R*_i_ (Ω) = *R*_IEM_ + *R*_feed solutions_ + *R*_el_ = *R*_AEM_ + *R*_CEM_ + *R*_LCC_ + *R*_HCC_ + *R*_el_ (4)

where *R*_LCC_ and *R*_HCC_ are the resistances of the low salinity and high salinity feed solutions, respectively. The potential of generated from RED (E) can be related to the current across the stack (*R*_i_) and loaded with a certain resistor *R*_L_, as follows:

*E*(*I*) = OCV − *R*_i_*I* (5)

In an ideal case, where no shortcut current flows in RED, *I* can be related to *E*, *R*_i_, and *R*_L_ as

(6) $$I\;=\;\frac{E}{R_{i}+R_{L}}$$

The power density, *P*_d_ (W/m^2^) of an RED connected to an external load resistance *R*_L_ can be calculated as

(7) $$P_{d}=\;\frac{I^{2}R_{L}}{2NA}=\;{\left(\frac{E}{R_{i}+\;R_{L}}\right)}^{2}*\frac{R_{L}}{2NA}$$

The maximum power density (*P*_d,max_) is obtained when *R*_L_ equals *R*_i_. Based on this assumption, combining Equations (5)–(7) leads to

(8) $$P_{d,\;\max}=\;\frac{OCV^{2}}{8AR_{i}}$$

where *A* (m^2^) is the active membrane area. The net output power (*P*_d,net_) is obtained by subtracting the hydrodynamic loss or the power loss over the pumps (*P*_h_) from the gross power density (*P*_d_):

*P_d,net_* = *P*_d_ − *P*_h_ (9)

provided that *P*_h_ is obtained from the theoretical pumping power consumed to circulate the solutions, which depend on the pressure drop over the HCC (Δ*P*_HCC_) (Pa), the LCC (Δ*P*_LCC_), and the volumetric feed flow rate *Q* (m^3^/s). Hence, Equation (9) can be re-written as

(10) $$P_{d,\;\mathrm{net}\;}=P_{d}-\frac{\Delta P_{\mathrm{HCC}}Q_{\mathrm{HCC}}+\Delta P_{\mathrm{LCC}}Q_{\mathrm{LCC}}}{NA}$$

### 2.2. Co-Ion Transport

Both experimental [20,21,22] and theoretical [15,21] studies were conducted in the literature to address the phenomena of water and ion transport through IEM. 

In RED, salt transport occurs by counter-ion (coulombic) transport and co-ion transport [20,22]. During ion transport in RED, the concentrations of the counter-ions within the charged membrane are much higher than the co-ions, as a result of the Donnan exclusion [3,22]. However, IEMs are somehow permeable for the co-ions as well. The co-ions are transported via the IEM from the HCC to the LCC in the same direction as the counter-ions. At zero current conditions, the transport of co-ions (i.e., NaCl diffusion via an IEM) can be compared with the self-discharge of normal electric batteries [21]. Studies demonstrated that co-ion transport was found to negatively impact the power density and efficiency of RED. A two-dimensional electromembrane model focusing on the effects of the co-ion transport in RED indicated a power density reduction of up to 20% [16]. Later in 2017, Tedesco et al. also demonstrated that co-ion transport either decreased the power efficiency, power density, or both at a given salt concentration [15]. Yip et al. demonstrated that the selectivity of typical IEMs (co-ion transport, osmosis, and electro-osmosis) lowered the efficiency of the process by up to 26% [22]. From the three transport mechanisms listed in this study, the co-ion leakage was found to have a dominant effect. The relative leakage of co-ions across the membrane can be quantified with a dimensionless parameter *β* [22],

(11) $$\beta =\;\frac{\Delta n_{s,\mathrm{co}}}{\Delta n_{s,\mathrm{ct}}}$$

where Δ*n_s_* represents the number of moles of salt transported across the IEMs and subscripts “co” and “ct” indicate co-ions and counter-ions, respectively. An ideal membrane has a *β* value of zero and allows only counter-ions, whereas a membrane with *β* = 1 allows the transport of both Na^+^ and Cl^−^ ions in equal pairs (i.e., a nonselective membrane). Membrane permselectivity (α) is related to β:

(12) $$\alpha =\;\frac{1-\beta }{1+\;\beta }$$

Mass balance of the flows into and out of the RED stack can be used to determine the transport of ions through IEMs. Thus, the total mass transport *T_m_* (mol/s) of NaCl from the HCC to LCC in the stack can be obtained as

(13) $$T_{m}={\emptyset_{d}}^{0}{c_{d}}^{0}-\;{\emptyset_{d}}^{i}{c_{d}}^{i}$$

where ∅ is the flow rate (m^3^/s) and *C* represents the concentrations (mol/m^3^) of feed. The superscripts “o” and “*i*” stand for “out” and “in”, respectively. The total mass transport results from two contributions, i.e., the counter-ions (coulombic part, T_coul_) and the co-ion part (T_cit_). The coulombic part is related to the *I* through the stack:

(14) $$T_{\mathrm{coul}}\;=\;\frac{I}{F}$$

The co-ion transport representing extra salt transport (T_cit_) can be determined as [20]

T_cit_ = T_m_ − T_coul_ (15)

### 2.3. Osmotic Transport

**Figure 2:**
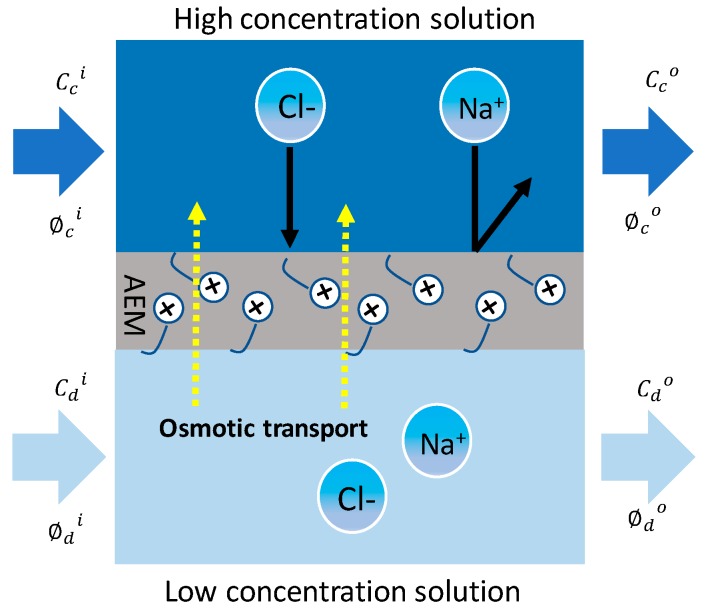
Transport in an AEM contacted with a NaCl feed solution.

In RED, the transport of water from the LCC to the HCC through an IEM causes osmotic transport [20]. The mechanisms that drive water transport through IEM involve osmotic transport (i.e., the osmotic pressure difference across the membrane drives the transport of water from LCC to HCC) and electro-osmosis (i.e., ions migrating due to an electric field drag nearby water molecules) [21]. The osmotic water transport and ionic diffusion are created by the presence of unequal concentrations of NaCl on each side of the membranes, which induces a potential chemical gradient over the membranes (Figure 2). The osmotic effect can be quantified based on the salt balance [20]:

(16) $${\emptyset_{c}}^{0}{c_{c}}^{0}+{\emptyset_{d}}^{0}{c_{d}}^{0}=\;{\emptyset_{d}}^{i}{c_{d}}^{i}+\;{\emptyset_{c}}^{i}{c_{c}}^{i}$$

Osmosis has a detrimental effect on RED by limiting the diffusion of the counter-ions through the IEMs and by diluting the boundary layer on the HCC side of the membrane [20]. It also reduces the thermodynamic efficiency of the RED. However, the negative effect of normal osmosis is counterbalanced by electro-osmosis and is less influential [22], but its negative effect to the same level as the co-ion transport was confirmed by model calculation [15]. It was also observed that the increase in the osmotic water flux reduced the membrane conductance [23] 

### 2.4. Electro-Osmosis

An electrostatic field created by the ions in the hydrated membranes drags the nearby polar water molecules. This leads to the phenomenon called “electro-osmosis” which is the transport of water molecuels with the ions in a direction opposite to the normal osmotic flow [20]. The advantage of electro-osmosis over co-ion transport and osmosis in RED is that it reduces the net water flux to the HCC [20,22]. However, in a typical RED operation, the net water transport from the HCC to the LCC solution due to electo-osmotic flux is lower than the osmotic flux [20]. 

### 2.5. Uphill Transport 

**Figure 3:**
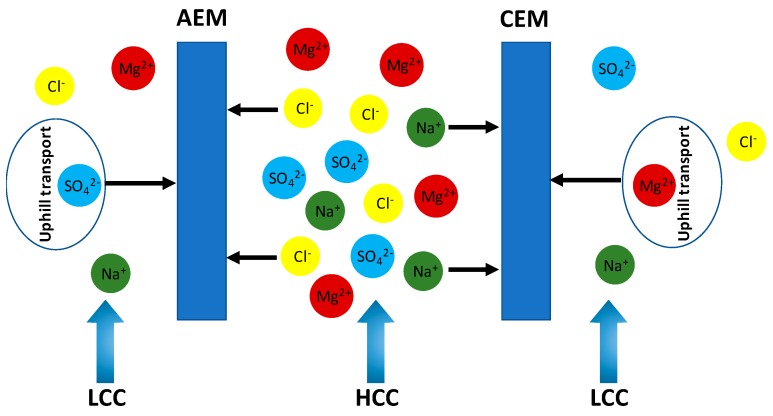
Illustration of uphill transport.

The presence of monovalent and multivalent ions on both sides of the IEM makes the transport phenomena very complex. Based on Equation (1), the voltage over each membrane due to each ion *I,* i.e., the Donnan potential (*E_i_*), is given by:

(17) $$E_{i}\;=\;\frac{\alpha RT}{Z_{i}F}\;ln\left(\frac{a_{i,c}}{a_{i,d}}\right)$$

where *a*_i_ is the activity of the ionic species. For instance, assuming a CEM separating artificial seawater and river water solutions containing mixtures of only NaCl + MgSO_4_ salts (Figure 3), and considering *a*_i_ of unity, then it appears from Equation (17) that the *E*_Na+_ (z = 1) is twice the voltage of *E*_Mg2+_ (z = 2). In other words, the salinity ratio of the monovalent ions creates twice the voltage generated by the same salinity ratio of divalent ions, assuming that the *a*_i_ and *α* remain constant. Under the OCV conditions, a higher voltage of Na^+^ forces Mg^2+^ to be transported from the river water to the seawater to maintain the electroneutrality that is disrupted due to the initial high transport rate of Na^+^ from HCC to LCC. This process is termed as uphill transport, in which two Na^+^ ions are transported in the opposite direction to the Mg^2+^ ions [5,6,7,8,24,25,26,27]. The Na^+^ and Mg^2+^ start moving until the overall *E*_i_ is balanced, reaching an equilibrium (*E*_Na+_ = *E*_Mg2+_); at this point, the uphill transport stops, defined as

(18) $$\frac{\alpha RT}{Z_{Na^{+}}F}\;ln\left(\frac{a_{Na^{+\;},c}}{a_{Na^{+},\;d}}\right)\;=\;\frac{\alpha RT}{Z_{Mg^{2+}}F}\;ln\left(\frac{a_{Mg^{2+\;},c}}{a_{Mg^{2+},\;d}}\right)$$

During uphill transport, the resulting voltage over the membrane is a value somewhere between the theoretical *E*_Na_^+^ and *E*_Mg_^2+^, i.e., less than the expected OCV for a pure solution of NaCl. This also leads to a reduction in the *P*_d_ of the RED process. Several studies were conducted to investigate the phenomenon of uphill transport in RED, even though a concrete justification to clarify the uphill transport and its impact on both the OCV and *P*_d_ of RED is lacking. Avic et al. demonstrated that uphill transport occurred in feed solutions (NaCl) with <30% in MgCl_2_ content [5]. Vermaas et al. revealed a substantial decrease in power density of up to 50% due to uphill transport when using total salt concentrations of 0.5 M in HCC solution and 0.017 M in LCC solutions, each with 10% MgSO_4_ and 90% NaCl solutions [8]. Losses in power densities of up to 29%–50% were reported in RED when using feed solutions with a 10% molar fraction of Mg^2+^ ion [28]. A faster transport of monovalent ions along with the concentration gradient is also favored when the temperature increases [5,6,25,29]. For instance, when increasing the temperature from 10 to 40 °C, the *P*_d,max_ of pure NaCl shows a larger increment (0.15 W/m^2^) than that of the NaCl–CaCl_2_ (0.10 W/m^2^) and NaCl–MgCl_2_ (0.11 W/m^2^) solutions due to the uphill transport of Ca^2+^ and Mg^2+^ [25].

## 3. Impact of Multivalent Ions at Different Salinity Levels

### 3.1. Impact under Low Feed Salinity Conditions

One of the challenges associated with RED application is the difficulty in working under realistic water resource conditions. Several of the reported research articles used model NaCl solutions at concentrations mimicking natural seawater and river water to assess the performance of RED [5,6,9,10,30,31]. However, natural sources (i.e., river water and seawater) contain a complex mixture of several ions (monovalent and multivalent) and natural organic matter, which jeopardize the performance of RED [5,32,33,34,35]. For example, an RED pilot plant equipped with ~50 m^2^ of IEM (125 cell pairs × 44 × 44cm^2^) produced 1.6 W/m^2^ of power density when the feed solution was natural brackish water (~0.03 M NaCl_equivalent_) and solar pond brine solution (4–5 M NaCl_equivalent_ + Mg^2+^ comprise up to 40% of solar pond brine) [33]. In contrast, by employing an artificial feed water solution (LCC: 0.03 M NaCl _equivalent_; HCC: 4–5 M NaCl_equivalent_) containing 3%–5% non-NaCl ions, a power output of 2.7 W/m^2^ with a ~60% increase in power density was reported [33]. This difference in power density was due to the presence of multivalent ions in the natural streams.

Table 1 presents the impact of divalent cations both at low and high feed salinity conditions. Under low feed salinity conditions (e.g., river water), the power output of RED is limited, mainly due to the rise in ohmic losses over the feed. For instance, the conductivity of river water (∼0.025 M NaCl, ∼2 mS/cm) is about 25 times lower than seawater (∼0.75 M NaCl, ∼49 mS/cm), and about 100 times lower than highly concentrated brine (∼5 M NaCl, ∼226 mS/cm) [3]. A very low concentration of the LCC solution or low conductivity results in high R_stack,_ which leads to low power density. Conversely, low concentration of the LCC solution increases the driving force which leads to high OCV. Therefore, a compromise between the R_stack_ and the OCV must be considered by optimizing the LCC solution concentration. The LCC solution concentration also depends on the operating conditions as well as the stack designs.

**Figure 4:**
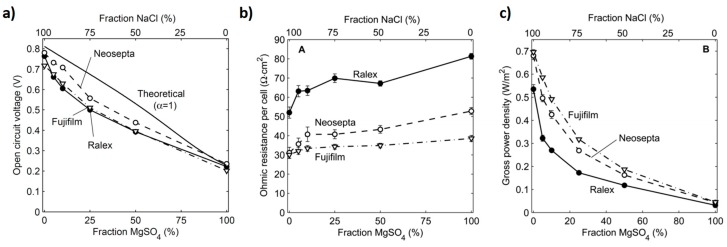
The impact of multivalent ions for RED systems tested using different commercial membranes (Ralex, Neosepta, or Fujifilm membranes). (**a**) Ppen-circuit voltage (OCV), (**b**) ohmic resistance, and (**c**) gross power density as a function of the molar fraction of MgSO_4_ of the total dissolved salts in the feed solutions. Experimental results are reported as an average of a stationary data series measured over 1 h. Reproduced with permission from [8]. Copyright 2015 Royal Society of Chemistry.

Several studies were performed to understand the impact of multivalent ions in an RED operated with feed solutions of very low concentrations. The decrease in membrane performance in RED (i.e., OCV, P_d max_,) due to the presence of divalent ions (SO_4_^2−^, Ca^2+^, and Mg^2+^) and natural organic matter (NOM) in various concentrations in real water were reported [34,35]. It was observed that inorganic solutes lowered the power density for the seawater/brackish water (SW/BW) and seawater/river water (SW/RW) water pairs by 8% and 4%, respectively [34]. In a similar study, Fontanova et al. tested mixtures of 0.34 M NaCl + 0.054 M MgCl_2_ and 0.5 M NaCl and observed about a 4-fold increase in the resistance of CEM (with sulfonic fixed charge groups) from about 2.41 Ω cm^2^ in pure NaCl to 8.3 Ω cm^2^ in the mixture [32]. The reason for the increase in resistance was mainly attributed to the lower mobility (due to higher hydrated radius) of Mg^2+^ than Na^+^, which contributed to the loss of permselectivity [5,8]. Mg^2+^ ions formed a bridge between two different fixed charged groups, reducing microchannels in the membrane and thereby restricting the ion [25,32]. Such a blocking effect was higher in the case of Ca^2+^ than Mg^2+^ due to the high binding affinity of the former toward the sulfonic fixed charged groups in CEM [25,32]. On the other hand, Mg^2+^ and Ca^2+^ displayed a negligible effect on the resistance of AEM due to the Donnan exclusion [32]. Vermaas et al. investigated the impact of multivalent ions using river water as a low salinity solution in RED [8]. As shown in Figure 4a, MgSO_4_ resulted in a reduction of OCV relative to the total amount of the dissolved salt in the feed water. As shown before, in a mixture of monovalent and multivalent ion solutions, the experimental OCV was lower than the theoretical due to the low Donnan potential. Permeation of co-ions via the IEM may also have occurred [5,8,25] (Figure 4a). Increasing the MgSO_4_ fraction increased the R_ohmic_ for all types of membrane due to the low conductivity of the feed water (Figure 4b). As the molar fraction of MgSO_4_ increased, the power density decreased for all types of membranes (Figure 4c). The reduction of the power density at 10% MgSO4 fraction when compared to pure NaCl feed solution was about 29% for Fujifilm, 37% for Neosepta, and 50% for Ralex membranes. This was coherent with the largest thickness and resistance of the Ralex membranes [8]. The low diffusion coefficients and mobility of the high ionic radii of the hydrated Mg^2+^ and SO_4_^2−^ contributed to the high resistance in RED [5,8].

In a mixture of NaCl/MgSO_4_ solutions with a molar ratio of 9:1 (a similar composition to the Mg^2+^ in seawater), with an HCC solution concentration of 0.508 M and an LCC solution concentration of 0.017 M, about 6% reduction in OCV was reported [36]. Up to 7.5% reduction in OCV was reported for RED operated under conditions of an LCC solution of river water (0.0153 M NaCl + 0.0017 M MgCl_2_) and an HCC solution of seawater (0.45 M NaCl + 0.05 M MgCl_2_) [26]. The impact of low feed salinity conditions was clearly seen when using 0.45 M NaCl + 0.05 M MgCl_2_ solutions in the LCC and 3.60 M NaCl + 0.40 M MgCl_2_ solutions in the HCC in RED stack [37]. Up to 20% and 60% reductions in OCV and P_d,_ respectively, were observed in the presence of 10% MgCl_2_ [5].This was associated with a 37% increase in the stack resistance. Model validation of experimental results was used to depict the negative impacts of divalent ions on RED performance [27]. Up to 16.3% reduction in power density was observed when using real solutions instead of pure NaCl solutions (HCC, 1 M Na+ and LCC, 0.02 M Na+) [27]. This reduction in power density was mainly attributed to the increase in the membrane resistance due to the presence of divalent ions in the test solutions.

### 3.2. Impact under High Feed Salinity Conditions

In RED, the use of highly concentrated solutions such as brine has the advantage of reducing the ohmic losses and hence increasing the OCV/power density. However, the highly concentrated solution also results in a decrease in permselectivity. Generally, permeselectivity or the effectiveness of co-ion exclusion of membranes is high in salt solutions with low concentrations of NaCl (˂0.5 M), but reduces dramatically when working with highly concentrated solutions (up to 5 M in NaCl) [29,40]. This mainly relates to the fact that the water flux dominates the salt flux when working with concentrated solutions. This was demonstrated by the work of Daniilidis et al., who determined the permselectivity of membranes in reverse electrodialysis operated with a pair of salt solutions with different concentrations [29]. They observed that membranes displayed lower permselectivity (˂90%) at high concentrations of the employed salt solutions (>0.5 M for the HCC solution and >2 M for the HCC solution). Combining an LCC solution with a high concentration (4 M) with an HCC solution of 5 M resulted in a decrease in permselectivity of up to 18.5% (corresponding to values below 50%). Permselectivities of up to 66% were reported by Tedesco et al. when using an LCC solution of 0.5 M NaCl and HCC solutions of 5 M NaCl in RED [40]. In fact, the use of highly concentrated solutions also runs the risk of membrane fouling such as scaling [3,38,41], which can be controlled by, for example, periodic air sparging and/or feed water reversal [42]. A high fixed-charge density of an IEM would benefit at high salinity gradients by reducing the permeation of the co-ions, whereas thick membranes would benefit at low salinity gradients by decreasing the osmotic flux.

**Figure 5:**
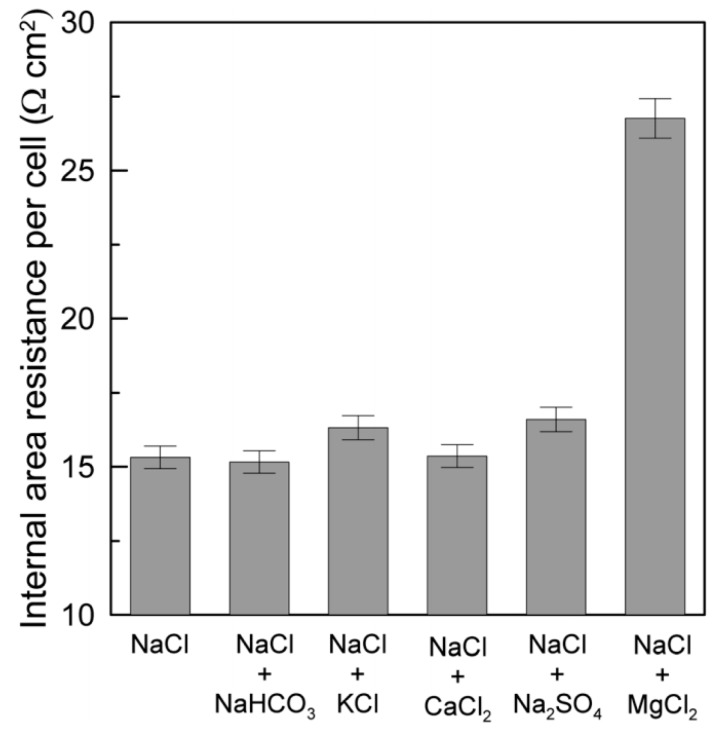
Variation in internal area resistance per cell with the composition of the feed solutions (single membrane area: 100 cm^2^; number of cell pairs: 25). Reproduced with permission from [9]. Copyright 2014 Royal Society of Chemistry.

**Figure 6:**
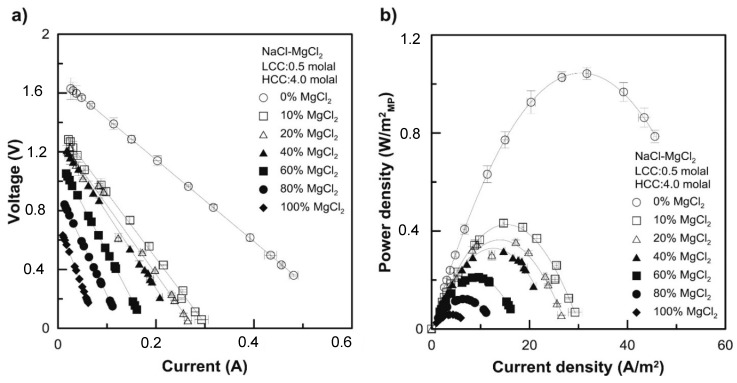
(**a**) Polarization curves (Voltage (V) vs. current (I)) and (**b**) gross power density *(P_d_)* as a function of current density for RED tests using multivalent ions (NaCl/MgCl_2_) of different molal compositions. In pure MgCl_2_ solution, the power density and the OCV decreased by 94% and 57%, respectively, with respect to pure NaCl solution. Reproduced with permission from [5]. Copyright 2016 Elsevier.

The undesirable impact of using feed solutions with high concentrations of multivalent ions on the performance of RED is huge [9]. Tufa et al. reported a reduction in power density of up to 63% accompanied by an increase in stack resistance of up to 76% when using feed solutions that mimicked real brackish water and brine instead of pure NaCl [9]. This reduction in the OCV and the power density was principally claimed to be due to the presence of Mg^2+^; other ions had a minor effect (Figure 5). This impact was more pronounced when a solution with increased concentrations of multivalent ions was used (Figure 6). [5,37]. For instance, 80% of MgCl_2_ in the feed solution decreased the OCV and P_d,max_ by up to 32% and 80%, respectively [5]. Also, 67% and 10% reduction in a power density and OCV respectively were reported when the feed concentration was shifted from an artificial (pure NaCl) to a natural (mixture) solution [6]. Avic et al. investigated the impact of Mg^2+^ in a lab-scale RED stack. A reduction in power density of up to 94% (from 1.06 to 0.06 W/m^2^) was reported when shifting from pure NaCl feed solutions (LCC: 0.5 M NaCl//HCC: 4 M NaCl) to multivalent ion solutions (LCC: 0.5 M MgCl_2_//HCC: 4 M MgCl_2_) [5]. This was associated with a 58% reduction in OCV (i.e., from 1.70 to 0.72 V). Moreover, the resistance was significantly affected by the presence of Mg^2+^. The stack resistance in the pure NaCl solution (LCC: 0.5 M NaCl//HCC: 4 M NaCl) was 2.78 Ω, which increased to 6.38 Ω in a mixture of solutions (LCC: 0.2 M NaCl + 0.3 M MgCl_2_//HCC: 1.6 M NaCl + 2.4 M MgCl_2_). The trend of the level of impact of multivalent ions on the maximum power density with respect to pure NaCl was Mg^2+^ > SO_4_^2−^ > Ca^2+^ ≈ K^+^ > HCO_3_^−^ [9].

In another study, the trend of divalent cations in increasing resistance of RED stack was shown to be Ba^2+^ > Ca^2+^ > Mg^2+^ [38]. The impact of Ba^2+^ was observed to be more pronounced due to its lower hydration radius of 4.04 Ȧ compared to Ca^2+^ (4.12 Ȧ) and Mg^2+^ (4.28 Ȧ), thereby leading to a strong electrostatic attraction by the fixed functional groups in the CEM and increasing the stack resistance. 

Generally, as the concentrations of divalent ions increase in the feed, remarkable decreases in the power density and the OCV and a substantial increase in the stack resistance are imminent. In general, further studies focusing on strategies to reduce both uphill transport and resistance are required to mitigate the impact of multivalent ions in RED stack. Developing a new generation of ion exchange membranes able to withstand the impact of multivalent ions should be envisaged. Other strategies, such as softening/pretreatment of the feed solutions, could also be considered when using highly concentrated brines in RED. Some of the strategies to alleviate the negative impact of multivalent ions in RED are presented in the following sections. 

## 4. Strategies to Alleviate the Impact of Multivalent Ions on RED

The existing challenges in RED can generally be advanced by employing novel materials and preparation techniques for commercial implementation. Often, membranes designed for RED have a slight difference than membranes applied for other electrochemical energy technologies [3], but this should not be the case, as the working environment might differ. For instance, in RED, the membranes are operated in nearly neutral pH conditions, in which Na^+^ and Cl^−^ are involved in ion transport. The desirable characteristics of IEMs for RED applications include low resistance, preferably designed without reinforcement, excellent permselectivity, and reasonable mechanical properties [2,11]. The key parameters that determine the performance of the RED are the resistance and the permselectivity of the employed membranes. The ideal membranes for RED should exhibit very low resistance below 1 Ω cm^2^ and high permselectivity above 95% [12,43]. Based on this, some strategies focusing on feed solutions or the employed membranes can be devised to reduce the adverse impact of multivalent ions on RED performance.

### 4.1. Feed Pre-Treatment 

Membrane filtration and chemical softening can be employed as a pre-treatment techniques for feed solutions in RED. Some of the nanofiltration [44] and ED membranes [44,45] can be used to separate monovalent and divalent ions. With ED stack, Zhang et al. reported that SO_4_^2−^ ion purity reached up to 85%, with a current efficiency of over 50% for NaCl/Na_2_SO_4_ mixtures [45]. ED with monovalent selective membranes was also used to separate divalent ions from seawater concentrate and enhance the purity of salt solutions. The results showed that low current density increased the selectivity of monovalent ions against divalent ions, with an optimal value of 4 mA/cm^2^. Recently, Rijnaarts et al. studied the possibility of removing divalent cations from freshwater using seawater as a draw solution in a Donnan dialysis (DD) process [46]. A 76% reduction in the divalent cation was achieved in natural freshwater, with a residence time of a few seconds. The DD pretreated freshwater showed improved gross and net power densities of up to 9% and 6.3%, respectively, in the RED process. However, introducing a pre-treatment (for feed pretreatment) step in RED is not attractive due to cost limitations [3,47].

### 4.2. Monovalent Selective Ion Exchange Membranes

**Figure 7:**
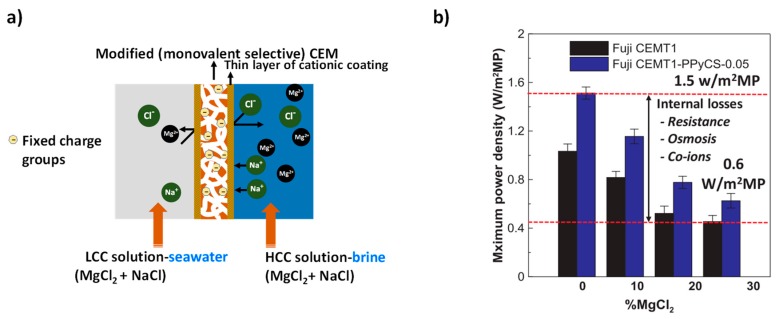
(**a**) Mitigation of uphill transport using monovalent selective cation exchange membrane. LCC: low concentration solution; HCC high concentration solution; CEM: cation exchange membrane. (**b**) Enhancement in OCV and power density with the monovalent selective membranes based on polypyrrole/chitosan composites. Reproduced with permission from [39]. Copyright 2016 Elsevier.

The development of a new generation of monovalent ion-selective membranes represents one of the key strategies to overcoming the problem of uphill transport in the RED process [24,26,48,49]. monovalent ion-selective membranes allow the passage of monovalent ions, but block the transport of multivalent ions (Figure 7a) [24,26,39,48,49]. Several factors, such as the differences in hydrated ionic radii, the differences in migration rate within the membrane phase, and the affinity of the ions with the membrane, affect the permselectivity between ions of the same charge [3]. Size exclusion and electrostatic repulsion are the two mechanisms that govern the mono/multivalent ions selectivity in RED/ED [39,48,49,50,51,52]. According to Coulomb’s law, electrostatic repulsion between multivalent anions/cations and a negative/positive surface potential is greater than that between monovalent anions/cations and a negative/positive surface potential [48,49,50,51]. This implies that multivalent anions/cations are less likely to be transported across the membrane than monovalent anions. The monovalent anion selectivity is attained by electrostatic repulsion created between the anions and the negative charge on the surface of the membranes [48,51]. 

The transport number ratio between mono- and divalent ions is used to measure the monovalent ion selectivity of the membrane [24,48,49]. After modification, the improvement of the monovalent ion selectivity of the membrane can be calculated as follows [48,49]:

(19) Pdiv mon= tmontdivCmonCdiv

where $t_{mon}$ and $t_{div}$ are the transport numbers of the mono- and the divalent ions, respectively, and $C_{mon}$ and $C_{div}$ are the average concentrations of the mono- and the divalent ions in dilute solution, respectively. A higher transport number ratio implies better monovalent selectivity, and vice versa [24,39,48,49,51,53].

Only a few studies focusing on the use of monovalent selective IEMs for RED applications are available [24,26,39,48,49]. Table 2 compares the monovalent ion selectivity of commercial (not specifically designed for RED) and tailor-made membranes. Generally, the transport number ratio of tailor-made monovalent ions selective membranes is higher than the standard/commercial monovalent selective membranes. However, in most cases, there is a trade-off between the enhancement of monovalent selectivity and membrane resistance when designing monovalent selective IEMs. As can be seen from Table 2, the resistance values of most of the commercial monovalent selective IEMs were higher than that of the tailor-made monovalent selective IEMs. The OCV of monovalent selective CMS (Neosepta) membrane remained comparable when using LCC solutions of (0.0153 M NaCl + 0.0017 M MgCl_2_) and HCC solutions (0.45 M NaCl + MgCl_2_ 0.05 M) instead of pure NaCl [26], indicating that the uphill transport of Mg^2+^ was effectively mitigated by using monovalent selective membranes. A comparative study of commercial IEMs, multivalent ion-permeable Fuji CEM T1 membranes, and monovalent selective Neosepta CMS membranes, was conducted for their performances in RED operated with multivalent ion solutions [26]. The use of Fuji CEMs T1 indicated a reduction in OCV when exposed to divalent-ion-containing feed solutions, whereas the CMS membranes were observed to be less prone to the reduction in stack voltage. However, a slightly higher power density was reported for Fuji T1 membranes than the Neosepta CMS membranes pertaining to its low membrane resistance. Gao et al. found that monovalent-ion selective poly(styrene sulfonate) (PSS)- and poly(ethyleneimine) (PEI)-modified AEMs (CJMA-2) showed an increase in power density of up to 17% more than the standard AEMs when using feed solutions containing Cl^−^, SO_4_^2−^, and natural organic matter (NOM) [48]. However, Moreno et al. revealed that the power densities of RED were not significantly improved by the use of highly cross-linked monovalent Neosepta CMS [24]. Uphill transport was mitigated, but the monovalent selective membranes experienced high resistance due to low ionic mobility of Mg^2+^.

Though both commercial and tailor-made monovalent ion selective membranes mitigate uphill transport, the use of a monovalent selective membrane may not be the ultimate solution for RED applications as the monovalent ion selective membranes have higher resistances than standard membranes, thereby resulting in lower power densities [49]. However, design strategies involving an insignificant change in membrane resistance could be envisaged to overcome this challenge. Tufa et al. reported an improved OCV and power density with pyrrole- and chitosan-modified CEM membranes in the presence of multivalent ions (Figure 7b) [39]. This improvement was mainly attributed to the insignificant change in membrane resistance during modification, although most studies showed insignificant changes in power density when using monovalent selective IEMs [24,49]. On the other hand, multivalent ion-permeable membranes with low resistances might also benefit from a reduction in the impact of multivalent ions, as shown earlier [24]. In general, further research is required to balance the high resistance created by the monovalent selective electrode with respect to the power output. 

## 5. Strategies for Developing Monovalent Ion Selective Membranes 

Some of the strategies for the design of monovalent selective IEMs include tuning of the membrane surface layer structure from dense and usually neutral polymers [48,53,54], surface layer modification with fixed ion exchange groups having opposite charges to those of the bulk membrane, and formation of a highly cross-linked surface layer with the same ion exchange groups as the bulk membrane 

### 5.1. Surface Modification

Surface modification of membranes can be employed to reduce electrical resistance while maintaining monovalent ion permselectivity. The membrane surface can be modified in such a way that high monovalent ionic flux and low electrical resistance are obtained. Physicochemical surface modification can be done by surface polymerization of polyaniline (PANI) [62], direct coating and electrodeposition [59], layer by layer (LbL) [51,53,54], and chemical modification. For instance, monovalent selective CEMs were synthesized from polyvinylidene fluoride (PVDF) and sulfonated-PVDF (s-PVDF) modified by PANI doped with p-toluene sulfonic acid (pTSA) or 2-amino-3-methylbutanoic acid (L-valine) [62]. During the performance tests in ED with feeds coming from Reverse Osmosis (RO) brine, membranes with PANI-pTSA ($P_{Na^{+}}^{Mg^{2+}}=0.13,\;P_{Na^{+}}^{Ca^{2+}}$ = 3.59) and L-valine ($P_{Na^{+}}^{Mg^{2+}}=0.09,\;P_{Na^{+}}^{Ca^{2+}}$ = 0.8) had higher selectivity for Na^+^ than the composite ones ($P_{Na^{+}}^{Mg^{2+}}=0.63,\;P_{Na^{+}}^{Ca^{2+}}$ = 6.82). Modification of commercial AEM by alternate electrodeposition of PSS/ hydroxypropyl trimethyl ammonium chloride chitosan (HACC) resulted in enhanced permselectivity of 2.90 (nine bilayers) compared to the pristine membranes with permselectvity of 0.66 [59]. A monovalent selective membrane was designed by coating the surface of a standard Fujifilm AEM by copolymerization of 2-acryloylamido-2-methylpropanesulfonic acid (AMPS) polymer and N,N-methylenebis (acrylamide) (MBA) as the active and the cross-linker, respectively [49]. The $P_{Cl^{-}}^{SO_{4}^{2-}}$ of the modified membranes was 0.755 (without a significant increase in the membrane resistance), which was higher than that of pristine membranes with a permselectivity of 0.841 [49]. The low sulphate flux of the modified membrane was due to its highly cross-linked structure and electrostatic repulsion [49]. Pan et al. used a one-pot approach to prepare internally cross-linked monovalent selective AEMs [60]. AEMs modified with sulfamerazine (SF) were synthesized by partial quaternization of chloromethylated (QPFS), followed by reaction with SF. At a pH of 6, higher permselectivity values $\left(P_{{\mathrm{SO}}_{4}^{2-}}^{{\mathrm{Cl}}^{-}}\right)$ of QPSF-SF-x (x = 0.05, 0.09, and 0.17 g), of 3.98, 15.9, and 3.28, respectively, than the pristine QPSF (1.28) membrane were reported. The excellent performance of the modified membrane was due to the fact that (i) SF creates a dense structure due to the cross-linking reaction between primary amine and the partial quaternization, and (ii) the sulfonamide group dissociated to form negative groups, therefore blocking the sulphate ions rather than chloride ions. However, at higher amounts of SF (i.e., 0.17 g), the permselectivity decreased due to the weak cross-linked structure, and the protonation of the unreacted SF protonated at pH 6 increased the positive charge in the membrane. The QPSF-SF-0.09 exhibited higher monovalent ion selectivity of 24.55 at pH = 10 compared to the commercial Neosepta ACS monovalent AEMs [60]. Recently, Tufa et al. prepared monovalent selective Fuji CEM T1 coated with pyrrole (i.e., 0.0025–1 M with a polymerization time of 1–8 h) and cross-linked with chitosan containing a weakly basic amino group at its end [39]. This modification led to membranes exhibiting a high degree of cross-linking with a thin cationic surface layer. Up to a 3-fold increment in monovalent selectivity was reported compared to the pristine membrane, mainly due to size exclusion (steric effects) and partly due to electrostatic repulsion [39]. Pyrrole and chitosan transformed the membrane structure into a rigid tight structure, resulting in restricted transport of Mg^2+^. Reductions in the degree of swelling, ion exchange capacity, and conductivity were also observed when modifying membranes using polypyrrole–chitosan composites with a high amount of pyrrole.

Physical attractions, such as electrostatic attractions and intermolecular forces (hydrogen bonds and Van der Waals forces), between the modified layer and the membrane matrix may result in a trade-off relationship between the permselectivity and the stability of the membranes [53,55]. For instance, in a Nafion membrane modified with PSS/PAH films consisting of 5.5 bilayers ((PAH/PSS)_5_PAH), the monovalent ion selectivity dropped rapidly because of the membrane instability at the interface of the active layer and the substrate layer [53]. In the LbL assembly, for example, as the number of bilayers increased, the monovalent selectivity increased until a certain level; afterward, the thickness and hence the membrane resistance increased [61]. An alternate electro-deposition of PSS/2-hydroxypropyltrimethyl ammonium chloride chitosan (HACC) on the surface of commercial AEM indicated sufficient monovalent selectivity for the 9-bilayer PSS/HACC-modified membrane [59]. However, the membrane resistance was observed to increase by almost 4-fold from 1.31 Ω cm^2^ to 4.52 Ω cm^2^. 

**Figure 8:**
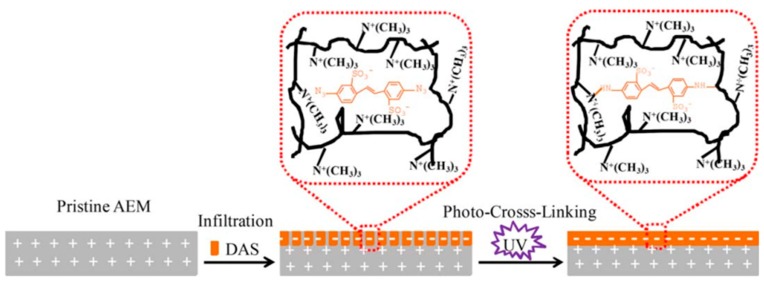
Surface modification of an anion exchange membrane (AEM) by infiltration and photo-cross-linking using 4,4-diazostilbene-2,2-disulfonic acid disodium salt (DAS). The azido group in DAS reacted to a nitrene group under UV irradiation which immobilized it on the membrane surface, thereby creating covalent cross-linking: The sulfonate group facilitated the water solubility and infiltration into the surface layer structure of the membrane, providing the negative charge groups and also improving the monovalent anion selectivity. Reproduced with permission from [57]. Copyrights 2018 American Institute of Chemical Engineers.

To overcome these challenges, other techniques, such as covalent immobilization of poly(ethyleneimine) (PEI) onto the surface of AEM [55], infiltration and cross-linking [57], and photoinduced covalent immobilization [58], were tested to form a stable linkage between the membrane and the modified layer. Covalent immobilization of PEI on AEM and partly quaternized poly(phenylene oxide) achieved excellent monovalent selectivity of ($P_{SO_{4}^{2-}}^{Cl^{-}}$ = 4.19) compared to the pristine one ($P_{SO_{4}^{2-}}^{Cl^{-}}$ = 0.79) [55]. During continuous cycling for 70 h in the ED process, the concentration of the sulphate ion was higher than the chloride ion, showing excellent monovalent ions selectivity and stability of the modified membrane. Very recently, Liu et al. modified AEM with 4,4-diazostilbene-2,2-disulfonic acid disodium salt [DAS] via infiltration and then covalent cross-linking using UV radiation [57]. As shown in Figure 8, the modified membrane had a negative charge toward the surface of the AEM, exhibiting the highest permselectivity of 11.21 compared with the commercial monovalent selective membrane (Selemion ASV) [49,63] and other reported modified AEMs (see Table 2) [48,49,55,58,59]. In an 80 h ED experiment, the modified membrane exhibited long term stability with constant selectivity.

Other research by Liu et al. showed the improvement of monovalent ion selectivity and the durability of AEM coated with polyelectrolytes [58]. Cross-linking via covalent bonds increased the stability of the polyelectrolyte. Commercial AEMs were also coated by alternating electrodeposition with polystyrene sulfonate (PSS) and HACC [58]. This multilayer was soaked with DAS solution to form a chemical bond under UV radiation. The monovalent ion selectivity of the modified membrane improved from 0.39 to 4.36 and stable selectivity of monovalent ions was observed for about 76 h during the ED operation [58]. CEMs (JAM-II-10) modified with surfactant N,N-dimethyl-N-2 propenyl-2-propene-1-1ammoniumchloride-2-propenamide(poly-quaternium-7, (PQ7)) resulted in a decreased leakage of Zn^2+^ from 22% to 14% during the ED test [64]. Good stability of the modified CEM was also observed due to the formation of sulfonamide bonds between the amine groups and the surface layer. 

### 5.2. Bulk Modification

Monovalent ion selectivity can also be achieved by bulk modification/morphology of the membrane networks [12]. Bulk modification involves the insertion of functional groups into the membrane matrix to improve certain characteristics of the IEM. This method can be achieved by blending two or more polymers in different concentration ratios to obtain the desired membrane properties [12,13,65]. Stability, permselectivity, and conductivity of IEM can be controlled by tuning the hydrophobic and hydrophilic properties of the blend membrane [66]. Tas et al. synthesized blend membranes based on a hydrolytically stable main-chain crown ether containing poly(arylene ether ketone) (CPAEK) and sulfonated poly(ether ether ketone) (SPEEK). This strategy was observed to enhance the selectivity of membranes for K^+^ ion over Li^+^ ion by a factor of ~4 [66]. Blending facilitated the hydrophobicity of the membrane along with the facilitation of the complexation reaction between the K^+^ and the crown ether.

Blaster et al. prepared and analyzed the separation properties of commercial monovalent CEMs and tailor-made monovalent membranes based on SPEEK and PES for solutions containing H^+^ and Ca^2+^ ions [67]. When the membrane conductivity and charge density increased, the Ca^2+^ permeation increased. Additionally, the Ca^2+^ flux increased when the Ca^2+^ concentration and/or current density increased. The conductivity and water uptake of the membrane decreased when the hydrophobic PES was blended with SPEEK [67]. By optimizing the amount of the hydrophobic PES blend and the degree of sulfonation in the SPEEK, high H^+^/Ca^2+^ selectivity was obtained while maintaining high membrane conductivity. Polymer blends can introduce specific complex-forming groups into the membrane, allowing adjustment of the final ionic flux and permselectivity of the membrane [66]. Ge et al. introduced acidic sulfonated poly(2,6-dimethyl-1,4-phenylene oxide) (SPPO-H) and basic 1-vinylimidazole (VI) monomer interactions into IEM to prepare the H^+^ selective membrane [68]. The poly(vinyl imidazole) (PVI)-SPPO network in the IEM was formed by polymerizing vinyl imidazole monomers in sulfonated PPO (SPPO) solution with PVI. With the increase in PVI, compact hydrogen bonds were formed in the membrane, thereby blocking Zn^2+^ leakage. On the contrary, electrostatic interaction between sulfonic acid with imidazole groups resulted in significant H^+^ ion flux via the formation of acid–base pairs in the membranes [68]. 

### 5.3. Layer-By-Layer Deposition

**Figure 9:**
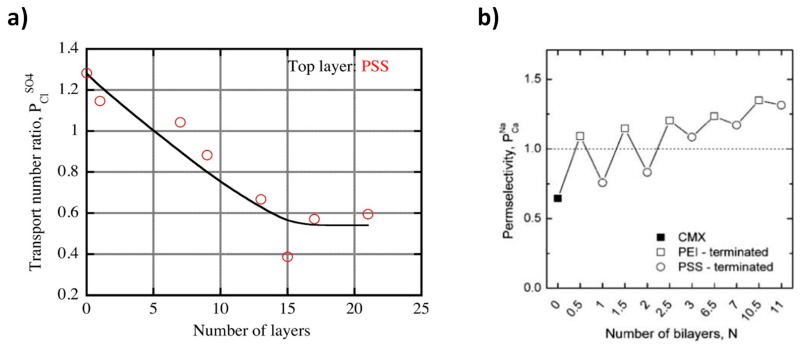
(**a**) The transport number ratio of SO_4_^2−^ and Cl^−^ ions in ED as a function of the number of layers for the AMX membrane modified with PSS end layers [51]. (**b**) Variations in the permselectivity of the Neosepta CMX with the number of PEI/PSS bilayers [54]. Reproduced with permission from [51,54]. Copyright 2013, Elsevier, and copyright 2014, American Chemical Society.

The LbL technique of polyelectrolytes is a facile method to design monovalent ion selective membrane [12,48,50,51,56]. LbL assembly is used for both CEM [53,54] and AEM [48,51,56,61] monovalent selective membranes (Figure 7). Instead of electrostatic deposition modification and electrodeposition modification, external driving forces, like the electric field and the electric pulse, are employed to assist LbL assembly to design homogenous and stable monovalent ion selectivity [48,56,61]. If no external force is employed, the LbL assembly of the polyelectrolyte is random, which may lead to the formation of excess charge within the multilayer thin film [48,56,61]. Goa et al. used LbL assembly to modify the surface of standard CJMA-2 AEMs by coating with PSS (for 20 min) and PEI (for 20 min) alternatingly [48]. Mulyati et al. used the PSS/PAH polyelectrolyte pair for the modification of Neosepta AEMs via LbL deposition [51]. Experiments under the ED test conditions (0.01 M NaCl and 0.01 M Na_2_SO_4_ feed solutions at a constant current density of 2 mA cm^−2^) indicated that the transport number ratio of the $P_{Cl^{-}}^{SO_{4}^{2-}}$ decreased with increasing layer numbers until the number of layers reached 15 (with PSS at the top), after which it remained constant (Figure 9a). The LbL deposition improved the monovalent ion selectivity, but a single layer deposition of PSS (Figure 9a) and PEI (Figure 9b) did not improve the monovalent selectivity. LbL deposition of PSS/PEI on CEM CMX Neosepta membrane resulted in monovalent selectivity at the 6th bilayer due to Donnan exclusion and hydrophobization [54]. Additionally, a qualitative indication of divalent ion repulsion of the membrane in a mixture of monovalent and divalent ions was observed due to double-layer capacitance.

Very recently, LbL assembly of AEM with NSBC/HACC at 7.5 bilayers showed improvement in monovalent ion selectivity due to the electrostatic repulsion and hydrophilicity of the modified surface (see Table 2 Reference [56]). LbL modification of commercial AEM was done by homogenizing hydrophilic poly(4-styrenesulfonic acid-co-maleic acid) (PSSMA) sodium salt and 2-hydroxypropyltrimethyl ammonium chloride chitosan (HACC) under an alternating current (AC) electric field, using 1,4 bis(2’,3’-epoxypropyl) perfluoro-1-butane as a cross-linker [61]. PSSMA with -SO_3_- had a negative charge, designed to become the innermost and outermost layer in the structure, and HACC had a positive charge, but both were hydrophilic. In ED experiment, the permselectivity of the modified AEM with AC∼LbL#7.5 was 4.87 compared to 0.81 for the pristine AEM [61]. After 96 h of ED operation, the AC∼LbL#7.5 AEM retained a permselectivity of 4.52. Furthermore, AC∼LbL #7.5 AEM indicated an excellent antifouling property [61]. 

During LbL assembly, the typical “odd–even” effect on ion selectivity resulted when the last surface layer ends with a polycation or polyanion [54]. The transport number ratio of $P_{Ca^{2+}}^{K+}$ of the modified CMX membranes increased with the PSS (polycation)/PEI (polyanion) number of layers (Figure 9b). However, the permselectivity of the modified membranes was enhanced when the LbL coating was terminated with PEI. The odd–even effect was stronger when there were less deposited layers and reduced with the number of bilayers. As shown earlier, LbL-modified Nafion membranes with 5.5 bilayers of PSS/PAH ((PSS/PAH)_5_PAH) showed a remarkable increase in $P_{Mg^{2+}}^{K^{+}}$ values from 22 to >1000 for [53]. However, after the 5.5 bilayers, the selectivity dropped because of the imminence of a stability issue. As the number of bilayer increased, the transport number ratio increased. When the PSS/PSI existed as 7.5 bilayers, the $P_{SO_{4}^{2-\;}}^{Cl^{-}}$ became 2.44, indicating that the monovalent selectivity of the modified AEM was highest at this bilayer ratio [48]. As the PSS/PEI ratio increased above 7.5, the $P_{SO_{4}^{2-}}^{Cl^{-}}$ started to decrease. 

In general, the advantages of monovalent AEM/CEM preparation by LbL include (i) the provision of a stronger Donnan exclusion of bivalent ions and (ii) the fact that the membranes mostly become hydrophilic/hydrophobic, depending on the end polyelectrolyte (i.e., polycation or polyanion). AEMs with hydrophilic surfaces were found to exhibit good antifouling properties [48,69]. The limitation of LbL deposition technique is that it is time-consuming to design membranes with the desired properties. In addition, as the number of deposited layers increases, the electrical resistance of the membranes increases. It was reported that a gradual decrease in permselectivity was observed during the continuous operation of RED (especially at high Mg^2+^ concentrations) due to the weak interactions of ionic polyelectrolytes and the functional group of the membrane [24]. 

## 6. Prospects in the Use of Conducting Polymers for RED

Design strategies that allow the preparation of membranes exhibiting high selectivity without compromising the resistance can be implemented by appropriately choosing base materials. Furthermore, most of the materials used to design monovalent selective IEMs for ED can be adopted for RED applications; the materials can be employed either directly or through modification in a certain way, for instance, using properly chosen materials to form composites. For example, the use of conducting membranes can be an interesting approach to design membranes exhibiting both low resistance and high selectivity. Conducting membranes, such as polypyrrole (PPy), exhibit attractive properties like good electrical conductivity and environmental stability, as well as easy synthesis [39,70]. Thus, this represents a highly attractive base material for the low-cost synthesis of novel materials for energy applications, including RED technology. Monovalent selective membranes can be designed with PPy as a base material or through modification with Pyrrole. Other conducting polymers, like polyaniline (PANI) and its composites, can also be promising materials to prepare monovalent selective IEMs. In addition to the use of intrinsically conductive materials for the design of monovalent selectivity, other strategies could involve design principles focused on multivalent ion-permeable membrane materials. In such a case, the effect of uphill transport would be compromised by the unrestricted transport of multivalent ions in the membranes leading to an insignificant change in the intrinsic membrane resistance. 

## 7. Other Prospects of Selective Ion Exchange Membranes 

Apart from RED, highly selective and conductive membranes are required in other electrochemical energy systems, such as fuel cells [71,72,73,74], water electrolyzers [75,76,77], alkaline batteries [78,79], and flow batteries [80,81]. Design strategies and base materials envisaged for the monovalent selective membranes in the present work can be systematically incorporated for applications in these technologies. For instance, LbL assembly can be used to endow monovalent ion selectivity for IEMs used in redox flow batteries [82,83]. Moreover, interesting works were reported regarding the modification of Nafion membrane by using polyelectrolyte LbL assembly to enhance proton conductivity while restraining vanadium ion permeability in vanadium redox flow batteries [82]. 

The monovalent ion selectivity and the permselectivity of the IEM itself are inter-related, meaning that both qualities of the membranes are inseparable during the optimal design of IEMs. For the best performance, the monovalent selectivity should be attained without affecting the permselectivity and resistance of the membrane. In line with the application of monovalent selective and/or permselective membranes for RED presented here, it is also important to note the applications of permselective membranes in other technologies. For instance, bipolar membranes, which consist of two ion exchange layers (AEM and CEM) of opposite charge in intimate contact, recently gained attention for applications in RED-based flow batteries [84], photoelectrochemical water splitting cells [85,86,87], and CO_2_ electrolyzers [88,89]. In electrochemical cells, bipolar membranes (BPMs) facilitate the water splitting at the membrane interface under reverse bias, driving OH^−^ to the anode and H^+^ to the cathode. This mechanism enables a constant pH to be kept on both sides of the cell, thereby allowing a more flexible system design. Under non-extreme pH conditions, the performance BPM of a bipolar membrane is influenced by the individual properties of the CEM and AEM layers [87,90]. In particular, the co-ion leakage, and therefore the permselectvity of the IEM layers, should be effectively directed toward designing highly efficient water and/or CO_2_ electrolyzers based on BPMs [88,90]. Overall, other membrane properties, such as conductivity and stability, also remain crucial in addition to membrane selectivity for some energy systems. Therefore, it would be important to clarify the key membrane requirements and structure–property relationships for the energy technology of interest before advancing to membrane design.

## 8. Conclusions and Outlook

In this work, the impact of multivalent ions on RED performance, the strategies used to alleviate such impact, and the design and use of monovalent selective IEMs were thoroughly reviewed. Different transport phenomena in RED, such as co-ions transport, electro-osmosis, water transport, and uphill transport were also discussed. These transport processes were reported to have detrimental impacts on the performance of RED. More importantly, uphill transport is one of the key challenges for the implementation of RED under natural feed salinity conditions with a mixture of monovalent and multivalent ions. The presence of both divalent cations and anions (Mg^2+^, Ca^2+^, SO_4_^2−^) in feed solutions imposed a significant negative effect on OCV and the power output of RED system. This effect is not well-understood, as the transport phenomenon in RED under conditions of mixture with monovalent and multivalent ion solutions is complex and requires advanced study through modeling approaches combined with experimental outputs. The effects of multivalent ions are generally quite variable when working with natural feed solutions containing a mixture of ions under high- and low-salinity conditions. Some strategies were reported to reduce the impact of multivalent ions in RED, such as feed pretreatment and the use of monovalent ion selective membranes. The use of monovalent selective IEMs may be preferable compared to the inclusion of a pretreatment step in RED, given that these membranes are designed with low-cost materials. Moreover, different strategies exist to design monovalent selective ion exchange membranes, and the choice should mainly be based on simplicity and the possibility of implementation for large-scale applications. For example, chemical modification methods would be more challenging compared to modification strategies based on UV irradiation, which are fast, simple, and promising for large-scale implementation.

The key challenge when designing monovalent selective membranes using new materials or by modifying commercial membranes is the existing trade-off between membrane selectivity and membrane resistance. The existing commercial monovalent selective membranes incorporating such strategies and materials in one way or another are not optimally designed for RED applications. Furthermore, only a few works reported tailor-made IEMs specifically designed for RED applications. Therefore, a gap in the research focused on designing highly selective membranes for the implementation of RED under natural feed salinity conditions still exists.

## Figures and Tables

**Table 1:** The impact of divalent ions on the performance of RED under low and high feed salinity conditions.

LCC (M)	HCC (M)	Membranes	α (%)	R_stack_	P_d, max_ (W/m^2^)	OCV (V)	Ref.
NaCl: 0.025	NaCl: 0.75	PC-SK and PC-SA	84	69.9 Ωcm^2^	0.32	1.393	[34]
^1^ BW: Na^+^ (0.024), Cl^−^ (0.032), SO_4_^2−^ (0.002), Ca^2+^ (0.00) Mg^2+^ (0.001), K^+^ (0.000)	^2^ SW: Na^+^ (0.390), Cl^−^ (0.578), SO_4_^2−^ (0.024), Ca^2+^ (0.027) Mg^2+^ (0.03), K^+^ (0.006)		67	86.5 Ωcm^2^	0.11	0.926	
^3^ RW: Na^+^ (0.004), Cl^−^ (0.008), SO_4_^2−^ (0.000), Ca^2+^ (0.000) Mg^2+^ (0.000), K^+^ (0.000)	^4^ SW: Na^+^ (0.39), Cl^−^ (0.578), SO_4_^2−^ (0.024), Ca^2+^ (0.027) Mg^2+^ (0.03), K^+^ (0.006)		72	150 Ωcm^2^	0.17	1.49	
^5^ GW: Na^+^ (0.059), Cl^−^ (0.093), SO_4_^2−^ (0.003), Ca^2+^ (0.001), Mg^2+^ (0.003), K^+^ (0.002)	^6^ RO: Na^+^ (0.269), Cl^−^ (0.409), SO_4_^2−^ (0.009), Ca^2+^ (0.004) Mg^2+^ (0.015), K^+^ (0.004)		78	46.7 Ωcm^2^	0.07	0.53	
NaCl (0.1)	NaCl (0.5)	Fuji-AEM-80045 and Fuji-CEM-80050	96	4.59 Ωcm^2^	-	-	[32]
NaCl (0.5)	NaCl (4)		99	5.68 Ωcm^2^	0.96	1.71	
NaCl (0.1)	NaCl (5)		89	7.58 Ωcm^2^	1.95	3.02	
NaCl (0.340) + MgCl_2_ (0.054)	NaCl (2.716) + MgCl_2_ (0.428)		56	17.3 Ωcm^2^	0.67	1.47	
NaCl (0.473) + MgCl_2_ (0.014)	NaCl (3.78) + MgCl_2_ (0.11)		63	8.58 Ωcm^2^	0.76	1.64	
NaCl (0.083) + MgCl_2_ (0.017)	NaCl (2.708) + MgCl_2_ (1.458)		33	54.2 Ωcm^2^	0.60	1.32	
NaCl (0.03)	Brine : NaCl (5) + 2–3% Non-NaCl ions	Fujifilm AEM RP1 80045-01and Fujifilm CEM RP1 80050-04	90	-	2.7	-	[33]
BW: NaCl (0.03) + K^+^, Mg^2+^, Ca^2+^, SO_4_^2−^	Brine: NaCl (4–5) + K^+^, Mg^2+^, Ca^2+^, SO_4_^2−^		90	-	1.6	-	
BW: NaCl (0.1)	Brine; NaCl (5)	Fujifilm-AEM- 80045 and Fujifilm-CEM- 80050	-	3.83 Ω	3.04	3.4	[9]
BW Na^+^ (0.066), Cl^−^ (1), SO_4_^2−^ (0.0035), Ca^2+^ (0.003) Mg^2+^ (0.0014), K^+^ (0.001), HCO_3_^−^ (8.3 × 10^−6^)	Exhaust brine: Na^+^ (2.9), Cl^−^ (4.8), SO_4_^2−^ (0.67), Ca^2+^ (0.006) Mg^2+^ (1.6), K^+^ (0.2), HCO_3_^−^ (0.0008)		-	6.76 Ω	1.13	2.77	
NaCl: (0.0999975) + NaHCO_3_ (8.5 × 10^−6^), [Cl^−^]/[HCO_3_^−^] = 11,717	NaCl: (4.99915) + NaHCO_3_ (8.5 × 10^−4^), [Cl^−^]/[HCO_3_^−^] = 5841		-	3.79 Ω	3.03	3.39	
NaCl: (0.098) + KCl (0.002), [Na^+^]/[K^+^] = 52.1	NaCl: (4.68) + KCl (0.32), [Na^+^]/[K^+^] = 14.5		-	4.08 Ω	2.84	3.4	
NaCl: (0.096) + CaCl_2_ (0.004), [Na^+^]/[Ca^2+^] = 26.4	NaCl: (4.99) + CaCl_2_ (0.01), [Na^+^]/[Ca^2+^] = 474		-	3.84 Ω	2.84	3.27	
NaCl: (0.0966) + Na_2_SO_4_ (0.0034), [Na^+^]/[SO_4_^2−^] = 28.8	NaCl: (4.39) + Na_2_SO_4_ (0.61), [Na^+^]/[SO_4_^2−^] = 7.15		-	4.15 Ω	2.79	3.40	
NaCl: (0.083) + MgCl_2_ (0.017), [Na^+^]/[Mg^2+^] = 4.99	NaCl: (3.25) + MgCl_2_ (1.75), [Na^+^]/[Mg^2+^] = 1.86		-	6.69 Ω	1.11	2.73	
NaCl (0.5)	NaCl: (4)	Fujifilm-AEM- 80045 and Fujifilm-CEM- 80050	-	2.78 Ω	1.06	1.70	[5]
NaCl (0.45) + MgCl_2_ (0.05)	NaCl (3.60) + MgCl_2_ (0.40)		-	4.44 Ω	0.43	1.36	
NaCl (0.40) + MgCl_2_ (0.10)	NaCl (3.2) + MgCl_2_ (0.80)		-	4.67 Ω	0.36	1.3	
NaCl (0.30) + MgCl_2_ (0.20)	NaCl (2.40) + MgCl_2_ (1.6)		-	5.11 Ω	0.32	1.29	
NaCl (0.20) + MgCl_2_ (0.30)	NaCl (1.60) + MgCl_2_ (2.40)		-	6.38 Ω	0.21	1.15	
MgCl_2_ (0.50)	MgCl_2_ (4)		-	8.92 Ω	0.06	0.72	
RW: Na^+^ (0.001), Cl^−^ (0.0005)	SW: Na^+^ (0.78), Cl^−^ (0.59)	Fujifilm-AEM- 80045 and Fujifilm-CEM- 80050	68	12.8 Ω	1.41	4.09	[6]
RW: Na^+^ (0.001), Cl^−^ (0.0005), K^+^ (0.0001), Mg^2+^ (0.001), Ca^2+^ (0.0038), SO_4_^2−^ (0.0001)	SW: Na^+^ (0.78), Cl^−^ (0.59), K^+^ (0.017), Mg^2+^ (0.088), Ca^2+^ (0.01), SO_4_^2−^ (0.027)		68	30.5 Ω	0.46	3.68	
RW: NaCl (0.017)	SW: NaCl (0.513)	Fujifilm-CEM-Type I and Fujifilm-AEM- type I	-	1.9 Ωcm^2^	-	-	[38]
RW: NaCl (0.017)	SW: NaCl (0.4617 + 0.02565 (MgCl_2_)		-	2.77 Ωcm^2^	-	-	
RW: NaCl (0.017)	SW: NaCl (0.4617 + 0.02565 (CaCl_2_)		-	3.29 Ωcm^2^	-	-	
RW: NaCl (0.017)	SW: NaCl (0.4617 + 0.02565 (BaCl_2_)		-	3.8 Ωcm^2^	-	-	
RW: NaCl (0.017)	SW: NaCl (0.5)	Fujifilm Type I AEM and homogeneous T0 CEM	-	-	-	1	[26]
RW: NaCl (0.0153) + MgCl_2_ (0.0017)	SW: NaCl (0.5)		-	-	-	0.966	
RW: NaCl (0.0153) + MgCl_2_ (0.0017)	RW NaCl (0.45) + MgCl_2_ (0.05)		-	-	-	0.925	
Pure NaCl: (0.5)	Pure NaCl (4)	Fujifilm-CEM T1	87–91	1.69 Ω/cm^2^	1	0.21	[39]
NaCl (0.35) + MgCl_2_ (0.15)	NaCl (2.8) + MgCl_2_ (1.2)		-	-	0.41	0.15	
Pure: NaCl: (0.008)	Pure: NaCl (0.5)	Fumatech-AEM-FKS-50 and Fumatech-CEM-FAS-50	0.92–0.96	-	1.6	-	[35]
RW: Na^+^ (0.008), Mg^2+^ (0.0014), Ca^2+^ (0.0014), SO_4_^2−^ (0.00026)	RW: Na^+^ (0.5), Mg^2+^ (0.056), Ca^2+^ (0.009), SO_4_^2−^ (0.03)		0.92–0.96	-	1.42	-	

^1^ BW: Brackish water; ^2^ SW: Seawater; ^3^ RW: River water; ^4^ SW: Seawater; ^5^ GW: Groundwater; ^6^ RO: Reverse osmosis.

**Table 2:** Comparison of the impact of monovalent ion selective membrane on the performance of ED and RED.

LCC	HCC	Membrane	A (%)	Δ (μm)	R (Ωcm^−2^)	OCV(V)	$P_{div}^{mon}$	Ref.
0.0153 M NaCl, 0.0017 M Na_2_SO_4_ 10 mg/L HA sodium salt	0.459 M NaCl, 0.051 M Na_2_SO_4_ 10 mg/L HA sodium salt	AEM-CJMA-2 multi	91.71	89	-	-	1.10	[48]
0.0153 M NaCl, 0.0017 M Na_2_SO_4_ 10 mg/L HA sodium salt	0.459 M NaCl, 0.051 M Na_2_SO_4_ 10 mg/L HA sodium salt	AEM-ACS mono	93.16	119	-	-	2.70	
0.0153 M NaCl, 0.0017 M Na_2_SO_4_ 10 mg/L HA sodium salt	0.459 M NaCl, 0.051 M Na_2_SO_4_ 10 mg/L HA sodium salt	^a^ AEM-CJMA-2 momo-TM δ	90.05	102.7	-	-	2.44	
RW: 0.012 M NaCl + 0.002 M Na_2_SO_4_	SW: 0.45 M NaCl + 0.05 M Na_2_SO_4_	AEM-Fuji A multi	89	123	0.93	1.01	0.841	[49] *
RW: 0.012 M NaCl + 0.002 M Na_2_SO_4_	SW: 0.45 M NaCl + 0.05 M Na_2_SO_4_	AEM-AMX multi	90	134	2.35	0.90	0.832	
RW: 0.012 M NaCl + 0.002 M Na_2_SO_4_	SW: 0.45 M NaCl + 0.05 M Na_2_SO_4_	AEM-ASV mono	96	110	3.07	-	0.730	
RW: 0.012 M NaCl + 0.002 M Na_2_SO_4_	SW: 0.45 M NaCl + 0.05 M Na_2_SO_4_	AEM-ACS mono	94	121	4.39	0.85	0.727	
RW: 0.012 M NaCl + 0.002 M Na_2_SO_4_	SW: 0.45 M NaCl + 0.05 M Na_2_SO_4_	^b^ AEM-Fuji A mono-TM	91	124	1.10	1.01	0.755	
NaCl 0.35 M + MgCl_2_ 0.15 M	NaCl 2.8 M + MgCl_2_ 1.2 M	Fuji CEM-T1 multi	87–91	117	1.69	0.15	-	[39]
NaCl 0.35 M + MgCl_2_ 0.15 M	NaCl 2.8 M + MgCl_2_ 1.2 M	^c^ Fuji CEM-T1 mono-TM	-	122	2.12	0.17	-	
0.05 M NaCl + 0.05 M CaCl_2_		CEM-CMX Neosepta standard	-	160	3.5	-	0.64	[54] **
0.05 M NaCl + 0.05 M CaCl_2_		CEM-CMS Neosepta mono	-	130	3.49	-	1.23	
0.05 M NaCl + 0.05 M CaCl_2_		CEM-CSO Selemion mono	-	90	4.09	-	1.72	
0.05 M NaCl + 0.05 M CaCl_2_		^d^ CEM CMX Neosepta mono-TM	-	-	-	-	1.24	
0.05 M NaCl + 0.05 M MgCl_2_		AEM-QPPO multi	-	-	4.63	-	0.79	[55] **
0.05 M NaCl + 0.05 M MgCl_2_		^e^ AEM- QPPO-PEI- mono-TM	-	-	5.30	-	4.19	
0.02 M NaCl + 0.02 M Na_2_SO_4_		Fuji AEM-T1 multi	-	125	1.31	-	0.81	[56] **
0.02 M NaCl + 0.02 M Na_2_SO_4_		AEM-ACS mono	-	120–200	3–6	-	13.6	
0.02 M NaCl + 0.02 M Na_2_SO_4_		AEM-ASV mono	-	120	3.1	-	22.3	
0.02 M NaCl + 0.02 M Na_2_SO_4_		AEM-AMX mono	-	120–180	2–3.5	-	-	
0.02 M NaCl + 0.02 M Na_2_SO_4_		^f^ Fuji AEM-T1 mono-TM	-	-	2.20	-	47.04	
0.05 M NaCl + 0.05 M Na_2_SO_4_		AEM-commercial multi	-	166	3.53	-	0.55	[57] **
0.05 M NaCl + 0.05 M Na_2_SO_4_		^g^ AEM mono-TM	-	-	4.50	-	11.21	
0.05 M NaCl + 0.05 M Na_2_SO_4_		Fuji AEM-T1 multi	-	125	1.30	-	0.39	[58] **
0.05 M NaCl + 0.05 M Na_2_SO_4_		^h^ Fuji AEM-T1 mono-TM	-	-	3.97	-	4.36	
0.05 M NaCl + 0.05 M Na_2_SO_4_		Fuji CEM-T1 multi	-	-	1.70	-	0.98	[52] **
0.05 M NaCl + 0.05 M Na_2_SO_4_		^i^ Fuji CEM-T1 mono-TM	-	-	3.93	-	5.1	
0.02 M NaCl + 0.02 M Na_2_SO_4_		Fuji AEM multi	-	125	1.31	-	0.66	[59] **
0.02 M NaCl + 0.02 M Na_2_SO_4_		^j^ Fuji AEM mono-TM	-	-	4.52	-	2.90	
0.05 M NaCl + 0.05 M Na_2_SO_4_ (pH = 6)		QPSF multi	-	-	2.86	-	1.28	[60] **
0.05 M NaCl + 0.05 M Na_2_SO_4_ (pH = 6)		^k^ QPSF-SF-0.05 mono-TM	-	-	3.19	-	3.98	
0.05 M NaCl + 0.05 M Na_2_SO_4_ (pH = 6)		^k^ QPSF-SF-0.09 mono-TM	-	-	4.04	-	15.90	
0.05 M NaCl + 0.05 M Na_2_SO_4_ (pH = 6)		^k^ QPSF-SF-0.17 mono-TM	-	-	7.89	-	3.28	
-		Fuji CEM-T1 multi	-	125	2.6	-	0.81	[61] **
50 mM Cl^−^ + 50 mM Sulphate		^l^AEM-AC∼LbL#1.5 mono-TM	-	∼125	3.18	-	1.42	
-		^l^AEM-AC∼LbL#3.5 mono-TM	-	∼125	3.88	-	2.11	
-		^l^AEM-AC∼LbL#5.5 mono-TM	-	∼125.5	4.94	-	3.71	
-		^l^AEM-AC∼LbL#7.5 mono-TM	-	∼125.5	6.88	-	4.87	

^a^ Layer by layer (LbL) deposition of poly(styrenesulfonate) (PSS) and poly(ethyleneimine) (PEI); ^b^ copolymerization of 2-acryloylamido-2-methylpropanesulfonic acid (AMPS) and N,N-methylenebis(acrylamide) (MBA); ^c^ polypyrrole/chitosan composite; ^d^ LbL modification with [(PEI/PSS)6 PSS]; ^e^ PEI-immobilized AEM with quaternized poly(phylene oxide(QPPO)); ^f^ LbL modification with N-O-sulfonic acid benzyl chitosan(NSBC) and hydroxypropyl trimethyl ammonium chloride chitosan (HACC) [(NSBC/HACC)7 HACC]; ^g^ infiltration and cross-linking of 4,4-diazostilbene-2,2-disulfonic acid disodium salt [DAS]); ^h^ alternating electrodeposition with polystyrene sulfonate (PSS) and 2-hydroxypropyltrimethyl ammonium chloride chitosan (HACC)-(PSS/HACC)_5_ PSS; ^i^ sandwich-like structure modification with upper/bottombilayers of polydopamine and sandwich alternating bilayers of poly(sodium 4-styrene sulfonates) (PSS) hydroxylpropyltrimethyl ammonium chloride chitosan nano silver particles (HACC-Ag-Np) – 4.5 bilayers; ^j^ alternate electrodeposition of poly(sodium 4-styrene sulfonate) (PSS) and hydroxypropyltrimethyl ammonium chloride chitosan (HACC)-(PSS/HACC)_9_; ^k^ sulfamerazine (SF)-modified AEMs partial quaternization of chloromethylated (QPFS)-QPSF-SF-x (x = 0.05, 0.09, and 0.17); ^l^ AC electric field, LbL modification of AEM with hydrophilic poly(4-styrenesulfonic acid-co-maleic acid) (PSSMA) sodium salt and 2-hydroxypropyltrimethyl ammonium chloride chitosan. (HACC) (AC∼LbL#n AEM (n = 1.5, 3.5, 5.5, and 7.5)); * the transport number ratio is calculated as $P_{mon}^{div}$; ** the performance of the membrane was tested in ED.^1^ RW: River water; ^2^ SW: Seawater; ^δ^ TM: Tailor-made.

## Abbreviations and Symbols

AEM	Anion exchange membrane
CEM	Cation exchange membrane
ED	Electrodialysis
HCC	High concentration compartment
IEM	Ion exchange membrane
LbL	Layer by layer
LCC	Low concentration compartment
NOM	Natural organic matter
P_d,max_	Maximum power density
R_non-omic_	Non-ohmic resistance
R_ohmic_	Ohmic resistance
OCV	Open-circuit voltage
*α*	Permselectivity
P_d_	Power density
RED	Reverse electrodialysis
RO	Reverse osmosis
SGP	Salinity gradient power
SWRO	Seawater reverse osmosis
R_stack_	Stack resistance
